# Claudin18.2 as a potential therapeutic target for primary ovarian mucinous carcinomas and metastatic ovarian mucinous carcinomas from upper gastrointestinal primary tumours

**DOI:** 10.1186/s12885-023-10533-x

**Published:** 2023-01-13

**Authors:** Fujun Wang, Yao Yang, Xiuzhen Du, Xiaoying Zhu, Yanjiao Hu, Changyu Lu, Lei Sui, Han Zhao, Kejuan Song, Qin Yao

**Affiliations:** 1 Department of Obstetrics and Gynecology, The Affiliated Hospital of Qingdao University, 16 Jiangsu Road, Qingdao, 266003 Shan Dong China; 2 Department of Gynecology, Junan County People’s Hospital, Junan, 276600 Shan Dong China; 3 Department of Gynecology, Qingdao Municipal Hospital, Qingdao, 266011 Shan Dong China; 4 Department of Pathology, The Affiliated Hospital of Qingdao University, Qingdao, 266003 Shan Dong China

**Keywords:** Ovarian mucinous carcinoma, claudin18.2, Primary, Metastatic, Upper gastrointestinal primary, Lower gastrointestinal primary

## Abstract

**Background:**

The vast majority of ovarian mucinous carcinomas are metastatic tumours derived from nonovarian primary cancers, typically gastrointestinal neoplasms. Therapy targeting claudin18.2 might be used in gastric, gastroesophageal junction and pancreatic cancers with high expression of claudin18.2. In this study, we aimed to profile the expression of claudin18.2 in primary ovarian mucinous carcinoma (POMC) and metastatic gastrointestinal mucinous carcinoma (MGMC).

**Methods:**

Immunohistochemistry was used to detect claudin 18.2 expression in whole tissue sections of ovarian mucinous carcinomas, including 32 POMCs and 44 MGMCs, 23 of which were derived from upper gastrointestinal primary tumours and 21 of which were derived from lower gastrointestinal primary tumours. Immunohistochemical studies for claudin18.2, SATB2, PAX8, CK7 and CK20 were performed in all 76 cases.

**Results:**

Among 76 primary and metastatic mucinous carcinomas, claudin18.2 was expressed in 56.6% (43/76) of cases. MGMCs from the upper gastrointestinal tract, including 22 derived from primary stomach tumours and one derived from a pancreas tumour, were positive for claudin 18.2 in 69.5% (16/23) of cases. MGMCs from the lower gastrointestinal tract, including 10 derived from primary appendiceal cancer and 11 derived from colorectal cancers, showed no claudin18.2 expression (0/21). The expression rate of claudin18.2 in primary ovarian mucinous neoplasms, including 22 primary ovarian mucinous carcinomas and 10 primary ovarian borderline mucinous tumours, was 84.4% (27/32). The common immunophenotypic characteristics of POMCs, upper gastrointestinal tract-derived MGMCs, and lower gastrointestinal tract-derived MGMCs were claudin18.2 + /PAX8 + /SATB2- (17/32), claudin18.2 + /PAX8-/SATB2- (16/23) and claudin18.2-/PAX8-/SATB2 + (19/21), respectively.

**Conclusion:**

Claudin18.2 is highly expressed in POMCs and MGMCs derived from upper gastrointestinal tract primary tumours; therefore, claudin18.2-targeted therapy might serve as a potential therapeutic strategy for POMCs and MGMCs from the upper gastrointestinal tract.

## Introduction

Ovarian mucinous carcinomas, including primary and metastatic cancers, account for 10 ~ 15% of epithelial ovarian cancers [1, 2], but primary ovarian mucinous carcinoma (POMC) represents only approximately 1 ~ 3% of epithelial ovarian tumours [1, 3]. Metastatic ovarian tumours may be derived from tumours in all parts of the body, and the most common primary sites are the gastrointestinal tract, breast, lung, and cervix, with gastrointestinal primary tumours, including upper and lower gastrointestinal tract tumours, being the most common origin [4, 5].

The 5-year survival rate of stage I POMC is 92%; however, if metastasis to the abdominal cavity occurs, in stage III and IV, the overall survival time is only 12–33 months, which is significantly lower than that of advanced-stage high-grade serous carcinoma (35 ~ 60 months) [3]. The prognosis of metastatic mucinous ovarian carcinoma is very poor, as patients usually die within 2 years of diagnosis; prognosis seems to be associated with the origin of ovarian metastatic carcinoma and optimal metastasectomy [6, 7]. The 5-year survival rates of metastasectomy-treated ovarian mucinous carcinoma originating from gastric, colorectal, and breast tumours are 0, 20.7 and 22.2%, respectively [7].

Since the morphological features of POMC and metastatic mucinous ovarian carcinoma are similar, differential diagnosis is difficult [8]. Furthermore, some clinicians suggest that oxaliplatin and 5-fluorouracil regimens are appropriate for postoperative treatment because primary mucinous carcinomas of the ovary are similar to gastrointestinal tumours [9]. The National Comprehensive Cancer Network (NCCN) guidelines recommend that POMC patients be treated with gastrointestinal malignancy chemotherapy regimens such as 5-fluorouracil and oxaliplatin, but their effectiveness has not been assessed in a large-scale study. Therefore, effective therapeutic approaches for primary and metastatic ovarian mucinous carcinoma deserve further study.

Claudin18.2 is highly expressed in gastric and oesophageal adenocarcinoma [10–12]. Claudin18.2 is a highly promising therapeutic target for claudin18.2-overexpressing tumours. Zolbetuximab (claudiximab), the first developed drug to target claudin18.2, is a chimeric IgG1 monoclonal antibody that specifically binds to claudin18.2 on the surface of tumour cells, triggering antibody-dependent cytotoxicity (ADCC), complement-dependent cytotoxicity (CDC), apoptosis and inhibition of cell proliferation. Preclinical studies successfully demonstrated that it has a powerful ability to clear cancer cells and control disease. Subsequently, its clinical efficacy and safety were evaluated by phase I/II/III trials [13–15]. Because the high specificity of claudin18.2 helps T cells recognize tumours, it is used for chimeric antigen receptor T-cell therapy (CAR-T). Patient-derived xenograft (PDX) models have been used to validate efficacy in vivo and thus may lead to breakthroughs, demonstrating claudin18.2-specific CAR T cells as a promising therapeutic strategy for claudin18.2-positive tumours, especially gastric cancer [16–18].

Claudin18 expression has been studied in borderline ovarian mucinous tumours and benign ovarian mucinous tumours [19, 20], but there is no study on claudin18.2 expression in ovarian mucinous carcinomas. Therefore, in our study, we investigated claudin18.2 expression in both POMCs and MGMCs to explore its potential role in targeted therapy.

## Methods

### Patient selection

Seventy-six cases of primary and gastrointestinal metastatic ovarian mucinous tumours were selected from Qingdao University Affiliated Hospital from 2013 to 2021. These included 22 POMCs, 10 primary borderline mucinous ovarian tumours, 21 lower gastrointestinal tract MGMCs (11 derived from colorectal primary tumours and 10 derived from appendix primary tumours), and 23 upper gastrointestinal tract MGMCs (22 derived from gastric primary tumours and 1 derived from a pancreatic primary tumour). Specimens were obtained for 20 cases of benign mucinous cystadenoma, 10 cases of benign ovarian serous cystadenoma, 10 cases of borderline ovarian serous tumours, 20 cases of primary ovarian serous carcinoma (10 cases of low-grade serous carcinoma and 10 cases of high-grade serous carcinoma), and 10 normal cases as a control (specimens were normal ovary and fallopian tube tissues from patients with uterine fibroids). For each specimen, the diagnosis was confirmed by two experienced pathologists according to the 2020 version of the World Health Organization (WHO) classification criteria, and none of the selected patients had received radiotherapy or chemotherapy before surgery. All study subjects gave informed consent, and the study protocol was approved by the Medical Research Ethics Committee of Qingdao University Affiliated Hospital.

### Immunohistochemistry

Specimens were fixed with 4% paraformaldehyde and embedded in paraffin, and specimen tissue wax blocks were continuously sectioned at 4 µm thickness. The slices were baked for 60 min in a 64 °C incubator. The sections were incubated 3 times in xylene for 5 min each to be completely dewaxed. The cells were incubated twice in 100% ethanol for 5 min each. The samples were hydrated by placing in 95%, 70%, 50%, and 30% ethanol for 2 min each. Antigen retrieval in citrate solution (pH = 6.0) was performed by heating in a pressure cooker to boiling, followed by spray steaming for 2 min. The sample was cooled in fine flow water to room temperature, rinsed with distilled water 2 times for 2 min each, and washed in phosphate buffered solution (PBS) 3 times for 5 min each. The samples were incubated with 3% hydrogen peroxide solution for 10 min and then washed in buffer for 20 min. Primary antibody was added, incubated at 37 °C for 1 h, and washed 3 times with PBS for 5 min each. Secondary antibody was added and incubated for 30 min at 37 °C in PBS 3 times, and each wash was performed for 5 min. diaminobenzidine (DAB) was added for colour development for 1 min and tap water for 2 min. The sample was stained in haematoxylin for 2 min and washed with tap water for 2 min. Then, 50%, 70%, and 95% ethanol were added for 2 min each, 100% ethanol was added 2 times for 2 min each, xylene was added 2 times for 2 min each, and a neutral gum sealing sheet was used. The stained slides were scanned with a Pannoramic SCAN slide scanner (3DHISTECH Ltd., Budapest, Hungary) to obtain a whole slide image. The primary antibodies were rabbit anti-claudin18.2 (clone ab222512, Abcam, United Kingdom, 1:500 dilution), rabbit anti-SATB2 (clone ab92446, Abcam, United Kingdom, 1:150 dilution), mouse anti-PAX8 (clone ab53490, Abcam, United Kingdom, 1:100 dilution), mouse anti-CK7 (clone ab9021, Abcam, United Kingdom, 1:500 dilution) and rabbit anti-CK20 (clone ab76126, Abcam, United Kingdom, 1:200 dilution).

### Scoring of immunohistochemistry

Claudin18.2 was expressed mainly in the cell membrane and partially in the cytoplasm, SATB2 and PAX8 were expressed in the nucleus, and CK7 and CK20 were expressed in the cytoplasm. Staining was assessed by two experienced pathologists using a double-blind method according to the proportion of positive cells (< 1% as 0, 1 ~ 25% as 1 + , 26 ~ 50% as 2 + , 51 ~ 75% as 3 + , and > 75% as 4 +) and the staining intensity (no colour is 0, weak is 1 + , moderate is 2 + , strong is 3 +). The product of the above two scores was taken as the final score. Negative expression was indicated by a score less than 4 + , and a score greater than or equal to 4 + indicated positive expression.

### Analysis of publicly available data

**Fig. 1:**
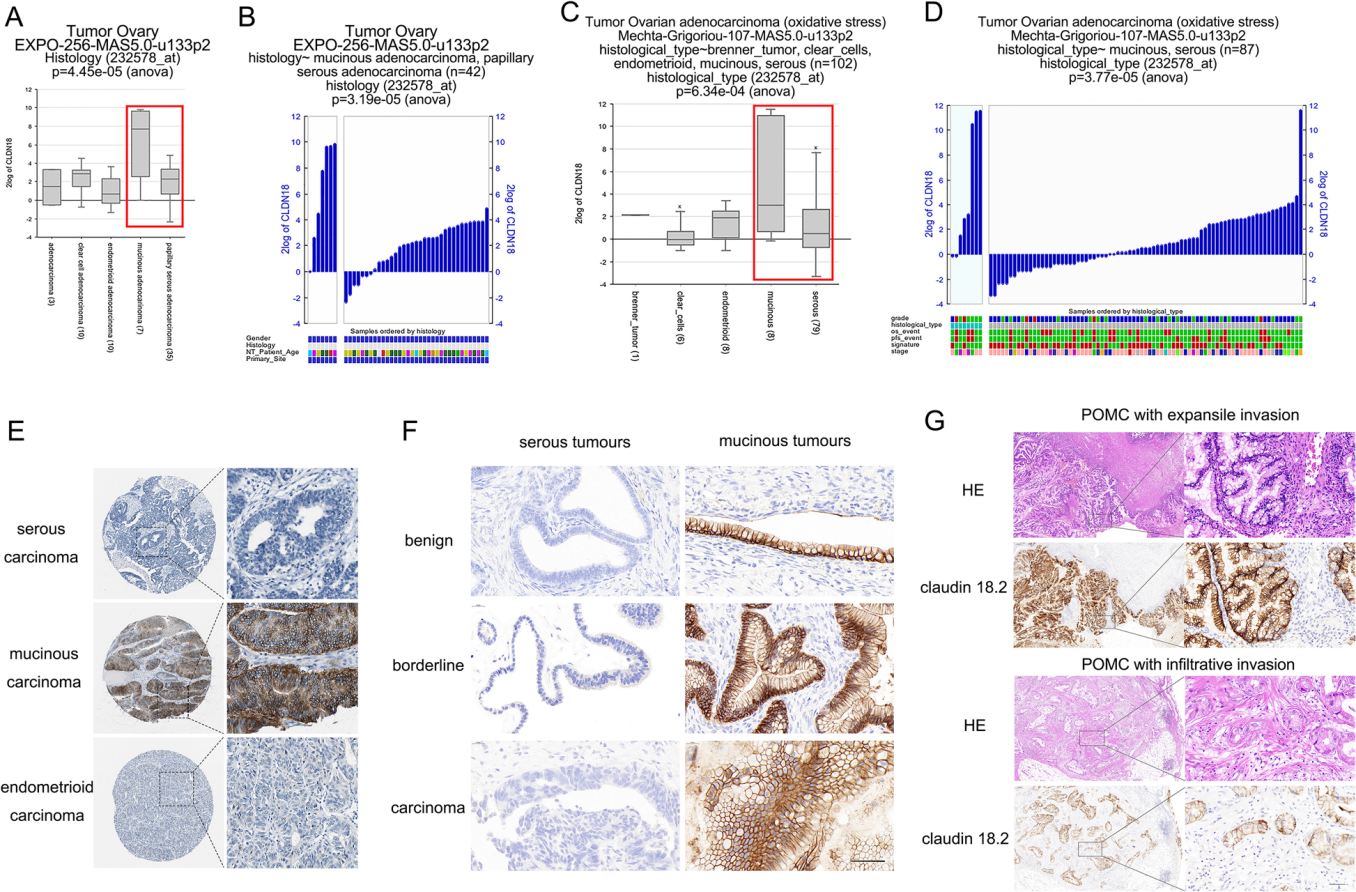
Claudin18 is upregulated in primary ovarian epithelial mucinous tumours. **A**, **C**. Box plot of claudin18 mRNA expression derived from publicly available data for human ovarian carcinoma samples. **B**, **D** Claudin18 expression in individual ovarian primary serous and mucinous carcinoma samples (highlighted in red box) (left panel = mucinous and right panel = serous). Figures were generated with the online R2 Genomics Analysis and Visualization Platform (https://hgserver1.amc.nl/cgi-bin/r2/main.cgi). **E** Claudin18 protein expression derived from publicly available data for human ovarian epithelial carcinoma samples, including serous, mucinous and endometrioid carcinomas. Data were from the HPA online platform (https://www.proteinatlas.org/). **F** Claudin18.2 expression in ovarian primary epithelial mucinous and ovarian primary epithelial serous tumours. Ovarian epithelial mucinous tumours (benign, borderline or carcinoma) were positive for claudin18.2 expression. Ovarian epithelial serous tumours (benign, borderline or carcinoma) were negative for claudin18.2. **G** Claudin18.2 expression in ovarian primary epithelial mucinous carcinoma with expansile or infiltrative invasion. (bar = 50 µm)

Claudin18 expression was analysed in patients with either primary or metastatic ovarian mucinous tumours (Fig. 1A-D). The RNA sequencing data are available in the Gene Expression Omnibus under accession numbers GSE2109, GSE26193, and GSE203611. We used the online R2 Genomics Analysis and Visualization Platform (http://r2.amc.nl) to visualize claudin18 gene expression data. To assess the expression of claudin18 protein in patient tissues of different pathological types of primary ovarian epithelial carcinoma, including serous carcinoma, mucinous carcinoma, and endometrioid carcinoma, we applied the HPA online platform (https://www.proteinatlas.org/).

### Statistical analysis

Statistical analysis was performed using SPSS 22.0 software. The statistical methods used were the χ2 test, Fisher’s exact test, nonparametric test, and t test. A *P* < 0.05 was considered statistically significant.

## Results

### Claudin18.2 expression in mucinous and serous primary ovarian tumours

The mRNA expression of claudin18 in primary ovarian epithelial malignancies is shown in Fig. 1A-D. The online R2 Genomics Analysis and Visualization Platform was used to visualize claudin18 gene expression based on RNA-sequencing data in GSE2109 and GSE26193, and the levels in mucinous adenocarcinoma were significantly higher than those in serous and endometrioid adenocarcinoma (Fig. 1A, C). Furthermore, the claudin18 mRNA expression levels in individual ovarian primary mucinous and serous adenocarcinoma samples were measured (Fig. 1B, D). Moreover, as shown in Fig. 1E, claudin18 protein expression was observed in ovarian primary epithelial malignancies, and staining was strong in the membrane and partially weak in the cytoplasm in mucinous cystadenocarcinoma. However, both serous carcinomas and endometrioid carcinomas showed negative claudin18 expression.

In whole tissue sections, claudin18.2 expression was negative in normal ovarian tissues and fallopian tube tissues. Mucinous cystadenoma had a positive expression rate of 85.0% (17/20), mucinous borderline tumour had a positive expression rate of 80.0% (8/10), and mucinous adenocarcinoma had a positive expression rate of 86.4% (19/22). Among POMCs, stage I/II mucinous adenocarcinoma had a positive expression rate of 80.0% (8/10) and stage III/IV mucinous adenocarcinoma had a positive expression rate of 91.7% (11/12), with an intensity ≥ 2 + in at least 50% of stained tumour cells. In 22 PMOCs, 6 cases were presented with infiltrative invasion; 1 case of stage IC and 5 cases of stage III had infiltrative invasion. And the claudin18.2 positive rates were 83.3% (5/6) in the POMCs with infiltrative invasion and 87.5% (14/16) in the POMCs with expansile invasion. All primary ovarian serous tumours, including 10 high-grade serous carcinomas, 10 low-grade serous carcinomas, 10 borderline tumours, and 10 benign tumours, showed negative claudin18.2 expression (Table 1, Fig. 1F, G).

**Table 1:** Expression of claudin18.2 in POMCs and MGMCs derived from the upper and lower gastrointestinal tract. POMC, primary ovarian mucinous carcinoma; MGMC, metastatic gastrointestinal mucinous carcinoma; UGI, upper gastrointestinal carcinoma; LGI, lower gastrointestinal carcinoma

Tumour type	Number	Positive expression	No labelling	Any labelling	Significant labelling (≥ 2 + in ≥ 50% of cells)	Significant labelling (≥ 2 + in ≥ 75% of cells)
Primary ovarian neoplasms						
Primary ovarian mucinous neoplasms						
Mucinous carcinoma	22	19(86.4%)	1(4.5%)	21(95.5%)	19(86.4%)	16(72.7%)
FIGO I-II	10	8(80.0%)	1(10%)	9(90.0%)	8(80%)	7(70%)
FIGO III-IV	12	11(91.7%)	0(0%)	12(100%)	11(91.7%)	9(75%)
Mucious borderline tumour						
FIGO I-II	10	8(80.0%)	2(20%)	0(0%)	0(0%)	8(80.0%)
FIGO III-IV	0	0(0%)	0(0%)	0(0%)	0(0%)	0(0%)
Mucinous cystadenomas	20	17(85%)	3(15%)	17(85%)	17(85%)	17(85%)
Serous neoplasms	40					
Low-grade serous cystadenomas	10	0(0%)	10(100%)	0(0%)	0(0%)	0(0%)
High-grade serous cystadenomas	10	0(0%)	10(100%)	0(0%)	0(0%)	0(0%)
Serous borderline tumour	10	0(0%)	10(100%)	0(0%)	0(0%)	0(0%)
Serous cystadenomas	10	0(0%)	10(100%)	0(0%)	0(0%)	0(0%)
MGMC						
MGMC from the UGI tract	23	16(69.6%)	1(4.3%)	22(95.6%)	13(56.5%)	7(30.4%)
Gastric primary	22	15(68.2%)	1(4.5%)	21(95.4%)	12(54.5%)	6(27.3%)
Pancreatic primary	1	1(100%)	0(0%)	1(100%)	1(100%)	1(100%)
MGMC from the LGI tract	21	0(0%)	21(100%)	0(0%)	0(0%)	0(0%)
Appendiceal primary	10	0(0%)	10(100%)	0(0%)	0(0%)	0(0%)
Colorectal primary	11	0(0%)	11(100%)	0(0%)	0(0%)	0(0%)

### Claudin18.2 expression in MGMCs derived from upper and lower gastrointestinal tract primary tumours

**Fig. 2:**
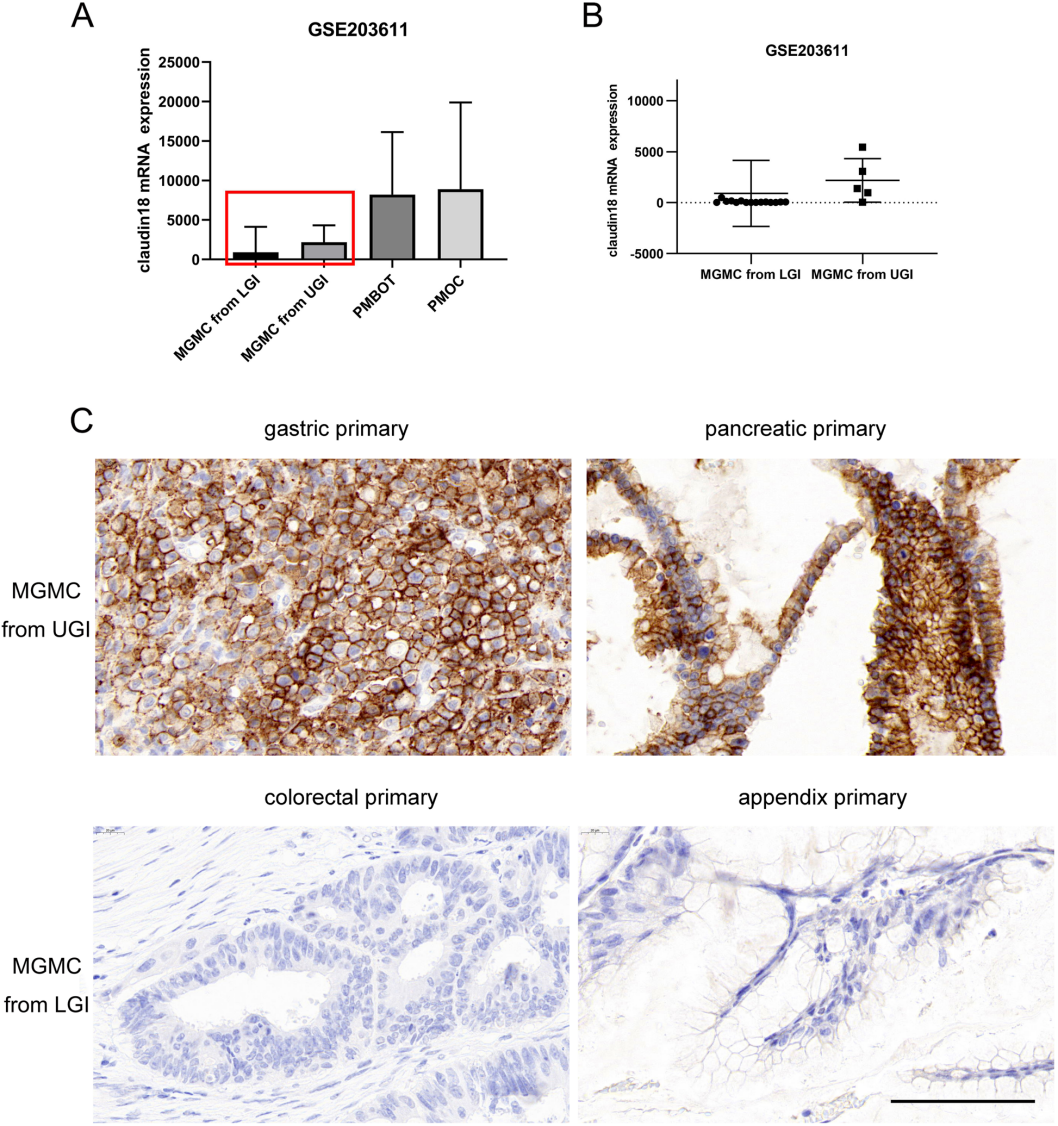
Claudin18 is upregulated in MGMC from the upper gastrointestinal tract. **A** Claudin18 mRNA expression derived from publicly available human ovarian primary mucinous ovarian tumours, MGMC in upper and lower gastrointestinal tumours. Data are available at the Gene Expression Omnibus under accession number GSE203611 (GEO Accession viewer (nih.gov)). Claudin18 expression in ovarian primary serous and mucinous carcinoma samples (highlighted in red boxes) is presented individually in (B). **B** Bar plot of claudin18 mRNA expression in MGMC in the upper and lower gastrointestinal tumours. **C**, High claudin18.2 protein expression in the MGMC of upper gastrointestinal tumours (gastric primary and pancreatic primary) and negative claudin18.2 expression in the MGMC of lower gastrointestinal tumours (colorectal primary and appendiceal primary). (bar = 100 µm)

As shown in Fig. 2A, B, the mRNA expression of claudin18 in ovarian metastatic mucinous tumours derived from the upper and lower gastrointestinal tract and primary ovarian mucinous tumours. Claudin18 expression levels were the lowest in metastatic ovarian mucinous tumours derived from lower gastrointestinal tract primary tumours and were significantly lower than in those of upper gastrointestinal origin.

In whole tissue sections, there were 23 cases of MGMCs of upper gastrointestinal origin, including 22 of gastric origin and one of pancreatic origin. The claudin18.2 positive rate was 69.6% (16/23), and 56.5% (13/23) of cases including 12 derived from primary gastric cancer and one derived from primary pancreatic cancer had an intensity ≥ 2 + in at least 50% of stained tumour cells. Labelling was localized to the membrane and partially accompanied by cytoplasmic staining. In contrast, a total of 21 MGMCs of lower gastrointestinal tract origin, including 10 from primary appendiceal tumours and 11 from primary colorectal tumours, did not show significant positive expression of claudin18.2 (Table 1, Fig. 2C).

### Immunohistochemical characteristics of claudin18.2, SATB2, PAX8, CK7 and CK20 in POMCs and MGMCs

#### POMC was compared with MGMC derived from upper and lower gastrointestinal tract primary tumours

The positive expression rate of claudin18.2 in POMCs was 84.4% (27/32), which was significantly higher than that in MGMCs derived from appendiceal and colorectal primary tumours (0/21) (*P* < 0.001), but not significantly different from that in MGMCs derived from the upper gastrointestinal tract primary tumours (69.6%,16/23). The positive expression rates of SATB2 in POMCs and MGMCs derived from upper gastrointestinal tract primary tumours were only 3.1% (1/32) and 4.3% (1/23), respectively, and the positive expression rate was up to 90.5% (19/21) in MGMCs from lower gastrointestinal tract tumours (*P* < 0.001). PAX8 expression was positive in 53.1% (17/32) of POMCs and not expressed in MGMCs (*P* < 0.001). The CK7 expression rate of POMCs was 96.9% (31/32), which was not different from that of upper gastrointestinal MGMCs (82.6%,19/23), but was higher than that of lower gastrointestinal MGMCs (33.3%,7/21) (*P* < 0.05). The CK20 expression rate of POMCs was 34.4% (11//32), which was lower than that of MGMCs derived from upper and lower gastrointestinal tract primary tumours (*P* < 0.05) (Table 2).

**Table 2:** Pathological phenotypic features of POMC and MGMC from the upper and lower gastrointestinal tract. *P* values indicating statistical significance are denoted. *p*1: POMC vs. lower gastrointestinal MGMC; *p*2: POMC vs. upper gastrointestinal MGMC; *p*3: upper vs. lower gastrointestinal MGMC; NS, no significant difference

	POMC(*N* = 32)	MGMC from the LGI tract (*N* = 21)	MGMC from the UGI tract (*N* = 23)	*p*_*1*_	*p*_*2*_	*p*_*3*_
Claudin18.2						
Positive	27(84.4%)	0(0%)	16(69.6%)	< 0.001	NS	< 0.001
Negative	5(15.6%)	21(100%)	7(30.4%)			
SATB2						
Positive	1(3.1%)	19(90.5%)	1(4.3%)	< 0.001	NS	< 0.001
Negative	31(96.9%)	2(9.5%)	22(95.7%)			
PAX8						
Positive	17(53.1%)	0(0%)	0(0%)	< 0.001	< 0.001	NS
Negative	15(46.9%)	21(100%)	23(100%)			
CK7						
Positive	31(96.9%)	7(33.3%)	19(82.6%)	< 0.001	NS	0.002
Negative	1(3.1%)	14(66.7%)	4(17.4%)			
CK20						
Positive	11(34.4%)	15(71.4%)	15(65.2%)	0.008	0.024	NS
Negative	21(65.6%)	6(28.6%)	8(34.8%)			

#### Comparison of MGMC from the lower gastrointestinal tract with MGMC from the upper gastrointestinal tract

Claudin18.2 showed negative expression in appendiceal and colorectal MGMCs; a significantly higher positive expression rate of 69.6% (16/23) was observed for gastric and pancreatic MGMCs (*P* < 0.001). SATB2 was observed frequently in MGMCs derived from the appendix and colorectum (90.5%,19/21), but was only observed in 1 case (4.3%) derived from the upper gastrointestinal tract (*P* < 0.001). PAX8 was not expressed in MGMCs derived from either upper or low gastrointestinal tract. The CK7 expression rate of MGMCs of upper gastrointestinal tract origin was 82.6% (19/23), which was higher than that of MGMCs with lower gastrointestinal tract origin (33.3%,7/21) (*P* < 0.05). The rates of CK20 expression in MGMCs of upper and lower gastrointestinal tract origin were relatively high [65.2% (15/23) and 71.4% (15/21), respectively], with no significant difference (Table 2).

**Fig. 3:**
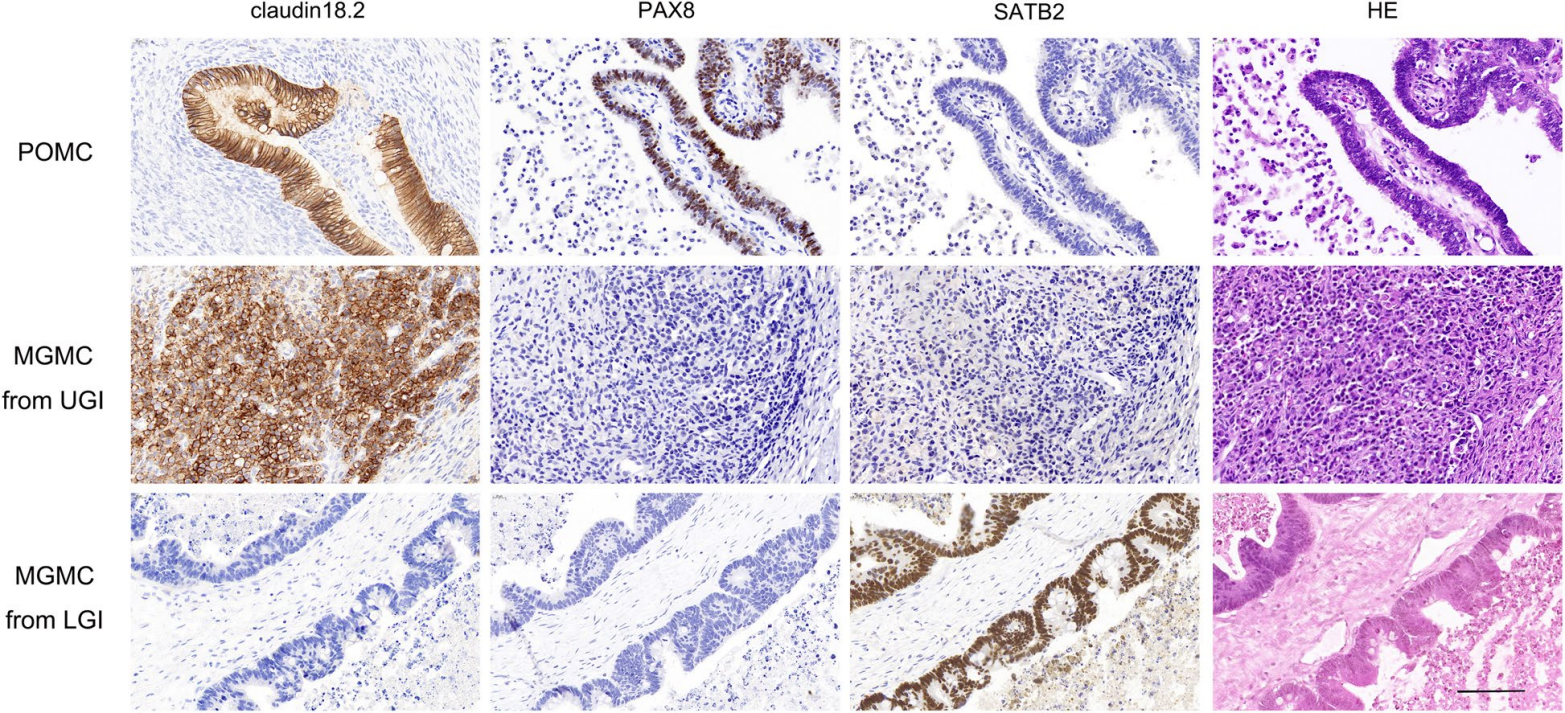
HE staining and staining for different immunophenotypic markers in POMCs and MGMCs from the upper and lower gastrointestinal tracts. The immunophenotypic characteristics of POMCs, upper gastrointestinal tract MGMCs, and lower gastrointestinal tract MGMCs were claudin 18.2 + /PAX8 + /SATB2-, claudin 18.2 + /PAX8-/SATB2- and claudin 18.2-/PAX8-/SATB2 + , respectively. (bar = 100 µm)

Therefore, the common immunophenotypic characteristics of POMCs, upper gastrointestinal tract-derived MGMCs, and lower gastrointestinal tract-derived MGMCs were claudin18.2 + /PAX8 + /SATB2- (17/32, 53.1%), claudin18.2 + /PAX8-/SATB2- (16/23, 69.6%) and claudin18.2-/PAX8-/SATB2 + (19/21, 90.4%), respectively (Fig. 3).

## Discussion

The SEER data from 1973–2015 showed that mucinous epithelial ovarian cancer patients exhibited better outcomes in the localized stage (FIGO I-A, I-B, I-not otherwise specified [NOS]) and regional stage (FIGO I-C, II-A, II-B, II-C, II-NOS) than in the distant stage (FIGO III and IV); for patients diagnosed at distant disease, the 3-, 5- and 10-year survival rates for mucinous epithelial ovarian cancer were 22.1, 16.1 and 10.9%, which were even worse than those for high-grade serous ovarian carcinoma (51.7, 31.9 and 15.5%) [21], which may be related to its poor response to chemotherapy and indolent biological behaviour [3, 22–24]. Improving the resection rate of advanced POMC can help to improve therapeutic efficacy [3]. Moreover, new strategies for the management of high-risk localized disease and advanced-stage POMC need to be assessed in clinical trials.

The prognosis of patients with gastric tumour-derived ovarian carcinomas was significantly worse than that of patients with colorectal tumour-derived ovarian carcinomas, and the survival time was 11 vs. 21.5 months [25]. Kajiyama et al. reported that 91.0% of patients with gastric tumour-derived ovarian carcinomas had recurrence, and 81.2% died of the disease during follow-up. However, 76.3% of patients with nongastric carcinoma developed recurrence, and 64.4% died of the disease. The median overall survival (OS) times were 8.1 and 28.1 months in patients with gastric cancer and nongastric cancer, respectively [26]. Among metastatic mucinous ovarian carcinoma types, those derived from the upper gastrointestinal tract have the worst prognosis, and effective therapeutics for these patients are worth exploring.

Claudin18 has two isoforms, claudin18.1 and claudin18.2, specific for pulmonary and gastric tissue, respectively. Claudin18.2 overexpression has been identified in several other types of cancers, including gastric, esophageal pancreatic adenocarcinomas [27–30]. In this study, we found a higher rate of claudin18.2 expression in POMCs and in MGMCs of upper gastrointestinal tract origin than in MGMCs of lower gastrointestinal tract origin. The positive expression rates of claudin18.2 in POMCs and borderline mucinous tumours were 86.4% and 80.0%, respectively. Positive expression was observed in 91.7% of advanced POMCs (FIGO III-IV). However, expression was negative in all ovarian serous tumours. In MGMCs, the positive expression rate of metastatic ovarian carcinomas from the upper gastrointestinal tract was 69.6%. Positive expression was observed included 15 cases of metastases from gastric cancer (15/22,68.2%) and 1 case of metastasis from pancreatic cancer (1/1), while no claudin18.2 expression was found in MGMC cases derived from the lower gastrointestinal tract.

Normally, claudin18.2 is expressed only on the surface of epithelial cells differentiated from the gastric mucosa, and claudin 18.2 expression in normal mucosal cells is restricted to the interior of the tight junction complex. However, after the occurrence of malignancy, the tight junction protein is destroyed, exposing the claudin18.2 epitope on the surface of the tumour cells, and making it an extremely ideal drug target.

Zolbetuximab(IMAB362) was the first mAb to target claudin18.2. In the FAST study, first-line treatment with zolbetuximab in gastric cancer led to a 50% increase in the progression-free survival (PFS) and a twofold increase in overall survival (OS) time [31]. Claudin18.2-positive gastric/gastroesophageal junction/oesophageal adenocarcinoma patients received first-line EOX (epirubicin plus oxaliplatin plus capecitabine) alone or combined with zolbetuximab. For patients with claudin18.2 expression in ≥ 70% of tumour cells, the median PFS with the zolbetuximab + EOX regimen was 9.0 months, and the median OS was 16.5 months. This group of patients benefitted most from zolbetuximab treatment. On the other hand, for patients with 40 ~ 69% claudin18.2 positive tumour cells, the median PFS with the EOX regimen was 5.7 months, and the median OS was 8.9 months [31]. In terms of efficacy, the higher the proportion of cancer cells expressing claudin18.2 is the more significant the advantage of the zolbetuximab regimen. Moreover, in the MONO study, zolbetuximab was used as monotherapy in patients with metastatic or progressive gastric/gastroesophageal junction/oesophageal cancer, including patients with moderate or strong claudin18.2 membrane staining in 50% of tumour cells. The regimen showed a 9% overall response rate (ORR) and a 23% clinical benefit rate (partial response and stable disease). In the subgroup of patients expressing claudin18.2 in ≥ 70% of the tumour cells, the ORR was increased to 14% [14]. Thus, the inclusion criterion for positive claudin18.2 expression in cancer cells was increased to 75% in many recent clinical studies [32]. In our study, a positive staining rate greater than 75% with a staining intensity score greater than 2 + was also applied as a critical value for assessing claudin18.2 expression. In the POMC group, 72.7% of patients had more than 75% claudin18.2-positive cells with moderate or strong staining, and 75.0% (9/12) of patients had in advanced-stage (stage III/IV) POMCs. For MGMCs of upper gastrointestinal tract origin, among 22 patients with tumours derived from gastric cancer, 6 patients (27.3%) had ovarian metastatic tissue with more than 75% stained tumour cells with intensity score ≥ 2 + . Moreover, one patient with pancreatic metastatic mucinous ovarian carcinoma had a score of 3 + in almost all tumour cells.

There are still limitations to this study. For both POMCs and MGMCs derived from upper gastrointestinal tract primary tumours, we should further compare claudin18.2 expression levels between primary and metastatic tumours. The combined detection of claudin18.2 and other targeted therapeutic targets, such as HER-2, PD-L1, MMR, and tumour-infiltrating lymphocytes (TILs), will facilitate the exploration of combined multitarget treatment strategies and the prediction of the response to these therapies for patients with POMCs and MGMCs.

According to the clinicaltrials.gov and chinadrugtrials.gov websites, dozens of clinical studies are currently ongoing to apply claudin18.2-targeted therapeutic strategies for solid tumours with high claudin18.2 expression. These claudin18.2-targeting agents include monoclonal antibodies (mAbs), bispecific antibodies (BsAbs), antibody-conjugated drugs (ADCs), and CAR-T immunotherapy. It is hoped that further clinical studies will confirm that patients with POMCs and MGMCs from the upper gastrointestinal tract with high claudin18.2 expression can benefit from therapeutic strategies targeting claudin18.2.

## Data Availability

The data used and/or analysed during the current study are available from the corresponding author on reasonable request.

## Abbreviations

POMC: Primary ovarian mucinous carcinoma
MGMC: Metastatic gastrointestinal mucinous carcinoma
NCCN: National Comprehensive Cancer Network
ADCC: Antibody-dependent cytotoxicity
CDC: Complement-dependent cytotoxicity
CAR-T: Chimeric antigen receptor T-cell
PDX: Patient-derived xenograft
WHO: World Health Organization
PBS: Phosphate buffered solution
DAB: Diaminobenzidine
PFS: Progression-free survival
EOX: Epirubicin plus oxaliplatin plus capecitabine
OS: Overall survival
ORR: Overall response rate
TILs: Tumour-infiltrating lymphocytes
mAbs: Monoclonal antibodies
BsAbs: Bispecific antibodies
ADCs: Antibody-conjugated drugs
